# Eating habits of preschool children and the risk of obesity, insulin resistance and metabolic syndrome in adults

**DOI:** 10.12669/pjms.306.5792

**Published:** 2014

**Authors:** Małgorzata Kostecka

**Affiliations:** Małgorzata Kostecka, University of Life Sciences in Lublin, Faculty of Food Science and Biotechnology, Lublin, Poland.

**Keywords:** Obesity, Nutritional imbalance, Metabolic syndrome, Children

## Abstract

***Background & Objective***: Nutrient excess and nutrient deficiency in the diets of preschool children can lead to permanent modification of metabolic pathways and increased risk of diet-dependent diseases in adults. Children are most susceptible to the adverse consequences of bad eating habits.The objective of this study was to evaluate the eating habits and the diets of preschool children as risk factors for excessive weight, obesity, insulin resistance and the metabolic syndrome.

***Methods***: The study was conducted on 350 randomly selected preschool children attending kindergartens in south-eastern Poland. Three-day dietary recalls were processed and evaluated in the Dieta 5 application.

***Results***: The analyzed diets were characterized by low diversity and a high share of processed foods, such as pate, sausages, ketchup, mayonnaise, fried meat, French fries and fast-food. The dietary content of vegetables, raw fruit, dairy products and whole grain products was alarmingly low.

***Conclusions***: Diets characterized by excessive energy value and nutritional deficiency can lead to health problems. In most cases, excessive weight gain in children can be blamed on parents and caretakers who are not aware of the health consequences of high-calorie foods rich in fats and sugar.

## INTRODUCTION

Nutrient excess and nutrient deficiency in the diets of preschool children can lead to permanent modification of metabolic pathways and increased risk of diet-dependent diseases in adults. 

Children are most susceptible to the adverse consequences of bad eating habits. An unbalanced diet can lead to delayed physical, cognitive and emotional development. Excessive supply of saturated fats and simple sugars combined with a deficiency of vitamins, minerals and highly available protein contributes to weight gain and glucose metabolism disorders. The results of numerous studies indicate that obesity in preschool children poses serious health risks and increases the prevalence of obese adolescents. 

Abdominal obesity increases the risk of insulin resistance 3-fold. A BMI greater than the 85 percentile increases the risk of unbalanced cholesterol levels 2.4-fold, unbalanced LDL cholesterol levels – 3-fold, unbalanced HDL cholesterol levels – 3.4-fold, unbalanced triglyceride levels – 7.1-fold, and hypertension – 4.5-fold.^1^

The objective of this study was to evaluate the eating habits and the diets of preschool children as risk factors for excessive weight, obesity, insulin resistance and the metabolic syndrome.

## METHODS

The study was conducted on 350 randomly selected preschool children attending kindergartens in south-eastern Poland. It was carried out in three stages between October 2013 and February 2014. In the first stage, children were subjected to anthropometric measurements, which involved the determination of height with the use of a stadiometer exact to 0.1 cm and body weight with the use of the Tanita BC 545N scales exact to 10 g. The measurements were used to calculate the body mass index (BMI) adjusted for age and gender in a centile chart (WHO).

The Cole index (CI), also known as the relative body mass index (RBMI), was calculated for every subject to determine the participants' weight relative to the average BMI at the 50^th^ percentile, with the use of the following formula:

Patient's BMI

RBMI = –––––––––––––––– x 100 [%]

 BMI at the 50th percentile

CI values are expressed as percentages (Table-I), and they are used to assess the nutritional status of children and adolescents.^2^

In the second stage of the study, parents were asked to fill out a questionnaire designed by the author to provide information about the family's eating habits, meal preparation methods, frequency of consuming various foods and knowledge about the nutritional requirements of preschool children. The parents participated in a 3-day dietary recall covering one day of the weekend. 

The children's preferences regarding food products and ready-made meals was evaluated in the third stage of the study. The subjects were presented with a picture-based questionnaire and were asked to mark different products with "like", "don't like" and "don't care" emoticons. The survey was carried out with the involvement of trained assistants who were students of the University of Life Sciences in Lublin graduating in nutritional sciences. 

**Figure:**
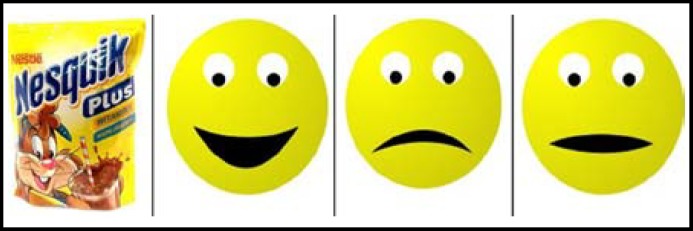


Three-day dietary recalls were processed and evaluated in the Dieta 5 application developed by the National Food and Nutrition Institute in Warsaw based on Polish food composition and nutrition tables.^3^^-^^5^ The average nutritional value and composition of the evaluated diets was determined in the Dieta 5 application. 

## RESULTS

The surveyed children were aged 4-6 years (average age 4.97 ± 0.84 years) and they attended public kindergartens. 58% of the surveyed subjects were girls. 5-year-olds had the highest share of the analyzed population.

According to the parents' declarations, the children's material and family situation was evaluated as satisfactory in 67% of cases, highly satisfactory in 21% of cases, unsatisfactory and unstable in 12% of cases. 

The results of anthropometric measurements revealed that 14.6% of all children (n=51) were overweight (Table-II). The majority of overweight subjects were girls (34), mostly 5-year-olds. The average RBMI was determined at 107.6% for girls and 103.3% for boys, where it did not exceed normal levels in any age group. Despite the above, not all boys were characterized by healthy body weight. The group of 4-year-olds included 3 boys with general obesity and RBMI values in the range of 123.5 – 131.2%.

The results of dietary recall interviews revealed that most children ate regular meals on weekdays, including 4 meals in the kindergarten and 2 meals at home. On weekends, 28% of the polled subjects ate only 3 meals daily, 8.8% children skipped breakfast and their first meal of the day was lunch served around noon. 95.4% children ate dinner, but the last meal of the day was served very late at 9 to 10.30 p.m.

The results of dietary recall interviews indicate that nutrient deficiencies (calcium, phosphorus, iron, vitamins E and D, unsaturated fatty acids and plant-based proteins) as well as nutrient excess (sodium, vitamin A, saturated fatty acids, simple and added sugars) were common in the analyzed diets (Table-III).

The average energy value of the analyzed diets was 1473.2 kcal ± 234.7, which was insignificantly above the recommended level. 

The evaluated diets were rich in high-calorie foods and added sugar. All children snacked between meals and had the highest preference for sweets and sweetened products. Sweetened fruit juice was consumed by 66% of the subjects, and sweetened soft drinks – by 44.6% of the children at least once a week. All children declared to have a preference for sweets: 203 subjects (58%) ate at least one serving of sweets per day, 118 children (33.7%) ate sweets several times a week, and only 8.3% of children ate sweets less frequently. The favorite products indicated by children were: candy, chocolate, biscuits, salty snacks, crisps, jelly sweets, nuts, crackers and fruit.

## DISCUSSION

The number of overweight children continues to increase and it poses a serious global problem.^6^^-^^9^Undesirable social behavior is the second most important contributor to excessive body weight after genetic factors. Parents and caretakers have very little knowledge about the impact of healthy nutrition on children's health and development. Overweight children are much more likely to become overweight and obese adults. Research results indicate that children aged 3 to 9 years with BMI above the 80^th^ percentile are three times more likely to suffer from obesity between the ages of 24 and 39, and the risk is four-times higher in overweight adolescents.^10^ According to the International Obesity Task Force,^11^ every fifth child in Europe is overweight. A Health Behavior in School-age Children (HBSC) study conducted in Poland in 2010 revealed that 18.3% of Polish adolescents aged 11-12 years were overweight and 3.4% were obese.^12^

**Table-I:** Classification of excessive body weight and obesity in children and adolescents based on the Cole index (CI).

**RBMI value**	**Nutritional status**
< 75%	Wasting
75–89%	Malnutrition
90–109%	Normal
110–119%	Overweight
>120%	Obesity

**Table-II:** Age structure and average RBMI values of the analyzed population

**Gender**	**Age **		
Girls [number of children]	4 years	5 years	6 years
	65	86	52
Average RBMI	106%	111.3%	105.5%
Number of overweight girls	7	23	4
Boys [number of children]	4 years	5 years	6 years
	37	67	43
Average RBMI	108.4%	99%	102.5%
Number of overweight boys	8	2	7

**Table-III:** Average concentrations of selected nutrients in the analyzed diets

	**Nutritional requirement**	**Average content in the analyzed diets ±** **standard deviation**	**Average content in the analyzed diets expressed in [%] of nutritional requirements**	***p***
Protein	45g	37.09 g ± 8.8	84.22	< 0.001
Carbohydrates	220g	194.7 g ± 87.9	88.5	< 0.001
including simple sugars	20-25g	140.3 g ± 63.8	561 -701	< 0.001
Total fat	45g	47.3 g ± 19.7	105.1	< 0.001
Saturated fat	15g	29.47 g ± 10.1	196.5	0.009
Monounsaturated fat	22.5g	13.78 g ± 7.4	61.24	< 0.001
Polyunsaturated fat	7.5g	4.05 g ± 2.2	54	0.004
Fiber	15g	11.9 g ± 4.7	79.3	< 0.001
Calcium	800 mg	553.25 mg ± 232.7	69.15	< 0.001
Magnesium	150 mg	166.5 mg ± 84.3	111	0.004
Phosphorus	1000 mg	641.6 mg ± 248.2	64.16	< 0.001
Iron	8 mg	6.64 mg ± 2.28	83	< 0.001
Sodium	1000 mg	2554.7 mg ± 358.4	255.47	< 0.001
Potassium	2100 mg	1552.7 mg ± 179.5	73.9	< 0.001
Vitamin A	2000 IU	2670 IU ± 291.1	133.5	< 0.001
Vitamin E	10 mg	4.02 mg ± 0.8	40.2	0.004
Vitamin C	50 mg	90.24 mg ± 47.2	180.48	< 0.001
Vitamin D	500 IU	234 IU ± 67.9	46.8	0.009
Folates	0.2 mg	0.06 mg ± 0.1	30	< 0.001

In the past decade, the incidence of obesity and the metabolic syndrome has increased at an alarming rate in South Asia, in particular among women and children. The main causes of obesity are economic development, changes in the traditional diet, lower levels of physical activity and genetic factors. In South Asia, the risk of metabolic and cardiovascular diseases has increased due to higher consumption of fat and abdominal fat deposition.^13^^,^^14^

Excessive weight gain during childhood and adolescence and abdominal fat deposition could be the first factors that increase the risk of the metabolic syndrome.^15^ In this study, 51 children were overweight, and most of them accumulated fat in the abdominal region or were characterized by general obesity. Snacking between meals and a preference for sweet-tasting foods can contribute to metabolic diseases such as hypertension, hyperglycemia, higher triacylglycerol (TG).^16^

Obesity prevention starting as early as in infancy is the most effective method of lowering the risk of the metabolic syndrome. Products rich in omega-3 and omega-6 fatty acids should be incorporated in the children's diet. In the surveyed population, the average consumption of unsaturated fatty acids covered nutritional requirements in 54% due to low consumption of fish. 45.1% of the polled children did not like and did not eat fish, whereas 17.1% of the subjects ate fish once a week, mostly in the form of fried bread-coated products. Body weight is also determined by the content of saturated fatty acids in the diet. In the analyzed population, the supply of saturated fatty acids accounted for 196% of nutritional requirements due to the consumption of highly processed fried foods. 211 children ate a hamburger at least once a week, 262 children – a slice of pizza, and 180 subjects – French fries as a serving of vegetables. 94.8% of the evaluated population consumed at least 15 g of hard cheese daily, which is a rich source of protein, but also fat. 

Other authors have demonstrated that high fast food intake leads to a three-fold increase in the risk of being overweight in children aged 6 to 11 years.^17^ Fast food is characterized by low nutritional value and a very high content of fat and saturated fatty acids.^18^

Those factors contribute to excessive body weight during childhood. The consumption of milk and dairy products that meet daily calcium requirements can inhibit the development of abdominal obesity in children aged 6 to 11 years. Several observational studies have demonstrated correlations between the consumption of dairy products and obesity in children, which indicates that milk protein plays an important role in body weight control.^19^ High and regular consumption of calcium, in particular from wholesome dairy products, can prevent excessive body weight and obesity.^20^ Milk and dairy product consumption was low in the studied population. 91 children (26%) did not like milk and did not consume milk in pure form or in milk soup. 180 children consumed 1 serving of milk or yogurt daily, and only 79 of subjects drank 500 ml of milk daily and ate other dairy products. The average calcium content of the analyzed diets covered only 69.19% of nutritional demand for this element.Similar results were reported by Uush, in whose study calcium intake in all age groups (1-3, 4-7, 8-12 years) was significantly below (39%, 30.9%, 24.4%) the recommended levels.^21^

Distribution of meals and regularity in eating are also important factors in body weight control. Breakfast avoidance can lead to excessive hunger, overeating,^22^ eating larger portions and excessive calorie intake in successive meals.^23^

 In the analyzed population, children tended to skip regular meals during the weekend. A late or skipped breakfast can disrupt appetite regulation. The consumption of sweet snacks at all times of the day further contributes to meal skipping and irregular meal patterns. Preschool children should eat 4-5 meals at regular times of the day. Regular meal patterns aid digestion and the utilization of nutrients and energy by the body.

The consumption of added sugars increases the energy value and lowers the nutritional value of a child's diet. Higher intake of simple sugars increases fasting blood glucose levels and impairs insulin secretion. Excessive supply of dietary glucose, fructose and saccharose can lead to blood sugar imbalance and insulin resistance.^24^^,^^25^

Simple sugar intake was very high in the analyzed diets, which contained 5-7 times more sugar on average than recommended for children aged 4-6 years. The above resulted from excessive consumption of sweets, juice, sweet soft drinks, sweetened tea and the children's general preference for sweet-tasting foods. Regular breakfasts consumption, higher intake of milk, oils rich in unsaturated fatty acids, fresh vegetables and fruit minimize the risk of excessive body weight, insulin resistance and the metabolic syndrome.^26^ Researchers and campaign authors havenoted a significant decrease in obesity, in particular among children aged 2 to 5 years (from 13.9% to 8.4%, p=0.03).^27^ In South Asia, Europe and the United States, which have the highest childhood obesity rates, the main objective of obesity prevention campaigns should be to increase awareness about diet-dependent diseases. There is a great need for community programs promoting physical activity and healthy eating habits among children.

## CONCLUSIONS

Irregular meal patterns and an unbalanced diet can impair physical and cognitive development in children. Diets characterized by excessive energy value and nutritional deficiency can lead to health problems. In most cases, excessive weight gain in children can be blamed on parents and caretakers who are not aware of the health consequences of high-calorie foods rich in fats and sugar. There is a dire need for social campaigns that promote healthy eating habits and prevent uncontrolled weight gain. In recent years, several healthy lifestyle campaigns in the US has given highly promising results. 
